# Utilization and Monetization of Healthcare Data in Developing Countries

**DOI:** 10.1089/big.2014.0053

**Published:** 2015-06-01

**Authors:** Joshua T. Bram, Boyd Warwick-Clark, Eric Obeysekare, Khanjan Mehta

**Affiliations:** ^1^ Humanitarian Engineering and Social Entrepreneurship (HESE) Program, The Pennsylvania State University, University Park, Pennsylvania.

**Keywords:** business intelligence, data acquisition and cleaning, data mining, data protection, privacy, and policy

## Abstract

In developing countries with fledgling healthcare systems, the efficient deployment of scarce resources is paramount. Comprehensive community health data and machine learning techniques can optimize the allocation of resources to areas, epidemics, or populations most in need of medical aid or services. However, reliable data collection in low-resource settings is challenging due to a wide range of contextual, business-related, communication, and technological factors. Community health workers (CHWs) are trusted community members who deliver basic health education and services to their friends and neighbors. While an increasing number of programs leverage CHWs for last mile data collection, a fundamental challenge to such programs is the lack of tangible incentives for the CHWs. This article describes potential applications of health data in developing countries and reviews the challenges to reliable data collection. Four practical CHW-centric business models that provide incentive and accountability structures to facilitate data collection are presented. Creating and strengthening the data collection infrastructure is a prerequisite for big data scientists, machine learning experts, and public health administrators to ultimately elevate and transform healthcare systems in resource-poor settings.

## Introduction

In many developing countries, efficient delivery of services is essential to provide citizens with adequate care. To accomplish this goal delivery, many ministries of health desire data about their populations to better understand the need they must fill. The director of the Brazilian National Health Service concluded that, “there is no healthcare without management, and there is no management without information.”^1^ This statement highlights the need for data to ensure both the efficient management and delivery of health services in low-resource environments. Data provides a quantitative basis for the deployment of resources. In developed countries, abundant amounts of data have driven healthcare decisions for many years with great success. In these countries, issues are related to practical applications of data analysis and machine learning techniques to harness these data in an actionable fashion. However, in developing countries, governments and organizations lack reliable systems for data collection, verification, and aggregation. Without systems in place that create and maintain robust, accurate, and relevant data, using data to address issues related to disease prevention, intervention assessment, and community education is just not possible.

Data collection and analysis programs to gather vital information have been attempted frequently. For example, Data for Development (D4D) in Cote d'Ivoire^2^ makes large data sets of healthcare data or telecommunications information available to the public. These are great opportunities for individuals to disseminate large quantities of data and draw important conclusions on populations. Additionally, conferences on data mining and articles on applied data statistics relating to the spread of disease,^3^ densification of electronic medical records,^4^ and management of resources are published every single year showing the potential of data for social good. However, large, reliable datasets are few and far between. This fact highlights an important issue—data collection in low-resource contexts is extremely challenging. While the diffusion of technologies like cellphones and laptops in developing countries has pushed governments and nonprofit organizations toward digital data collection and aggregation efforts, contextual, business, communication, and technological issues all stand in the way of collecting accurate and reliable data in the volume that is needed. Without data collection at its foundation and sustainable business models in place to ensure continuous aggregation of data, it is impossible to draw statistically sound conclusions when evaluating various initiatives. Reliable data collection is especially important as these organizations begin to look beyond impact assessment and toward prediction, specifically in the targeting of new areas, the estimation of future successes, and the personalization of treatments. These more complex analyses can be conducted using large datasets and machine learning techniques, but the quality of insight is purely dependent upon both the quantity and quality of data.

In fact, substantial opportunities surround the business potential of large data sets, something that only a few organizations and companies have harnessed to date. Large organizations like IMS Health and USAID invest millions of dollars every year for the acquisition of data across the world so that it can be analyzed and disseminated for the benefit of society. They work with local partners on the ground to overcome some of the largest contextual barriers to the efficient collection of data, again investing further time and resources. Only after the actual data is acquired can they release data that is suitable for effecting real social change through machine learning and data analytics.

To accomplish this difficult mission of data collection, many nations utilize community health workers (CHWs) to collect data during their routine check-ups on their neighbors. CHWs are usually government-trained volunteers that serve a set community of individuals (ranging in size from several hundred to several thousand per CHW), providing routine health consultations as well as triaging to medical clinics for treatment.^5^ CHWs also participate in the regular collection of patient health data in many nations. Because of their close ties to the community members and their roles as respected, conscientious individuals, CHWs serve as ideal agents for data aggregation and digitization. However, past efforts have shown that data collection efforts in conjunction with CHWs, who are unpaid volunteers, are not always effective.^6^ Instead, it has been found that further incentives must be provided to the CHWs for the consistent aggregation of data, as data collection represents an additional time burden that takes away from daily duties as well as other income-generating activities. Unfortunately, there are few practical business models in which CHWs are appropriately compensated for their data collection activities.

While CHWs serve as local champions for the collection of health data, it is at regional and national levels that healthcare data is often most important. This data is vital for the development and implementation of new policies. At this stage, governments, nonprofits, and even pharmaceutical companies stand to gain the most from comprehensive health data. The data serves to inform decision making, influence the direction of research efforts, and also provide information on the potential expansion of certain medications. These entities also present a logical source of cash flow to fund the collection of data that they will then utilize for their operations.

This article describes potential applications of health data in developing countries to improve community health and inform policy decisions. Subsequently, the diverse challenges to the collection and aggregation of data are discussed. Four business models that address these challenges by providing incentive and accountability structures are presented. These models provide a source of income for the CHWs collecting data, thereby ensuring the long-term sustainability of data collection efforts. These efforts are crucial to enabling continuous improvement driven by data.

## Applications of Patient Health Data

Many nonprofits, governmental organizations, and pharmaceutical companies are striving to identify methods of advancing healthcare by leveraging comprehensive population datasets. These groups base their efforts almost entirely on data that is made available publicly or data that they aggregate themselves. This section describes some of the most important applications of health data in developing countries.

### Identification of health trends and epidemics

At a regional or national level, it is important for policy makers to be informed of health trends so that they can be better informed when making decisions. For example, health data can be used to identify regional health trends like tracking the spread of drug resistance. OpenMRS, an online open platform software, allows the creation of summary graphs and searchable databases that have been used to track the resistance of *M. tuberculosis* to drugs in Peru before, during, and after treatment.^7^ This project has allowed nonprofits as well as over 220 medical clinics to gain access to information on TB resistance so that they can make more informed decisions about the applications of medications to which TB may be resistant.^8^

In 1998, several African nations adopted an integrated disease surveillance (IDS) strategy, showing a commitment to the aggregation of health data to better track diseases and epidemics.^9^ This included case-based surveillance so that countries could better track tropical diseases at a local level. In Uganda specifically, implementation of the IDS strategy led to a 30% increase in the reporting of disease cases from clinics, which was then used to identify the spread of diseases in the country.

### Tracking of immunization coverage

Immunization of individuals in an attempt to reduce the heavy burden of disease is a common strategy in both developed and developing countries. This preventive form of healthcare is easily managed in countries with strong infrastructures; however, in resource-poor settings, up-to-date information on the number of vaccinated individuals is vital to reducing the spread of many diseases such as polio and MMR. As a result of the challenges of vaccination, the WHO and UNICEF created the Global Immunization and Vision Strategy (GIVS) in 2006 to address the many difficulties associated with widespread immunization campaigns so as to bring developing countries closer to achieving the Millennium Development Goals.^10^ One of the four strategic foci of the GIVS was the integration of immunization efforts and other health interventions with surveillance so as to strengthen health systems.^11^ Another initiative, which was introduced in Africa in 2002, was the Reaching Every District (RED) strategy. This project also had the goal of increasing immunization coverage to reduce disease rates. One of its original five operational components was the monitoring and use of data for action, as coverage data provides reliable information on progress toward a program's target. The prevention of diseases and reliance on data to determine the modes of action for programs led the Polio Eradication Initiative to provide community health staff across the world with transport, communication, and data collection tools to better track polio vaccinations.^11^

### Targeting of health research efforts and interventions

The African continent is the epicenter of many prominent disease outbreaks such as Ebola and HIV. As a result, research studies by foreign governments and aid institutions are commonplace. However, in order to perform such studies, there is a requirement for reliable data on disease prevalence.^7^ It is also important to utilize focused health data for disease interventions, particularly with diseases such as HIV, where transmission is often a result of modifiable social behaviors. In Benin, along with much of HIV-endemic Africa, interventions targeted at female sex workers and the promotion of condom use have been particularly effective and have received increasing attention. These interventions are thought to be crucial for an eventual decline in the spread of HIV/AIDS.^12^

### Allocation of healthcare resources

Perhaps one of the most important uses for health data on a larger scale is the allocation and distribution of healthcare resources. Data can be used to better inform decision making and for improving health outcomes in communities, as it can detail the areas that are suffering the most from health-related issues.^13^ In Peru, an electronic medical record program allowed the government to not only detect problems, but also allocate resources to the areas hit hardest by TB.^7^ In a study in South Africa, a group of collaborators attempted to determine how to best allocate health resources by examining the socioeconomic, demographic, and physical characteristics of households in a community, all factors that are highly correlated with deprivation and ill-health.^14^ This allowed them to gain a better understanding of how governments and aid groups can allocate resources and represents a strategy that was entirely data driven. Because health resources are ultimately what reduce the incidence of disease, data that can better inform the targeting of such resources represents a crucial resource in itself, particularly in regions with limited supplies and finances.

### Education of healthcare professionals

Health data can also be used to improve the training of healthcare professionals. Keen knowledge of health data can give healthcare professionals a greater understanding and appreciation of actual health trends and treatment initiatives in place. Community-based health metrics can help health workers improve the quality of their assistance through recurring trainings. These tactics result in better delivery of healthcare and fewer incidences of disease. Specifically, specialty CHW programs exist to deal with and treat certain diseases such as HIV/AIDS, TB, or malaria.^5^ These specialist programs were piloted and driven by data collected from communities that demonstrated specific disease-based needs such as a high prevalence of HIV/AIDS.

### Management of staffing requirements

Another application of health data collected from patients is for use in better planning of health worker staffing requirements at hospitals and clinics. By utilizing patient health data to determine disease loads in particular communities, organizations can eliminate wasted finances that occur due to poorly allocated staff. An example is in Tanzania, where 99,000 full-time equivalent healthcare workers will be needed in 2015 based upon disease incidence, but only 37,000 are projected to be available.^15^ This illustrates the widespread lack of adequate healthcare staff across much of Africa and will allow the government to distribute staff to communities that suffer the most from specific diseases so that the few workers that are available are not underutilized.

### Reduction of wait times and increased patient volume at local clinics

Health data is critical to improving the care that patients receive, and in regions where health facilities are exceedingly uncommon, data and electronic records can be utilized to reduce the wait times for patients at clinics. In Mosoriot, Kenya, researchers implemented an electronic medical records system that allowed the aggregation of patient data for community members who visited the clinic. As a result of the new system, care providers (nurses and doctors) spent 58% less time with patients, which allowed those health workers to fit in more consultations each day.^16^ Patients also saw a 38% reduction in their wait times to see care, meaning that their healthcare delivery was both more efficient, and it allowed those individuals to return to their daily jobs or roles as caregivers sooner.

## Challenges to the Collection, Aggregation, and Digitization of Health Data

Despite the utility and potential of health data, it is often difficult to collect, aggregate, and digitize data in developing countries. Difficulties can be traced back to a wide range of issues, rooted in contextual, communication, business, and technological challenges.^17^ CHWs, in their role as trusted caregivers, present a unique opportunity to collect reliable data. Accordingly, several methods, including Internet-based forms, smartphone, or tablet device applications, voice-recognition software, manual transcription from paper forms, and text messages, have been attempted. Each of these approaches comes with its own set of limitations from a technological standpoint. Inconsistent power supplies often plague developing nations, so backing up large quantities of data can prove to be a challenge. Internet connectivity must also be reliable, accessible, and secure. The accuracy of the collected data can be questionable at times, with error rates as high as 4.5% for SMS.^13^ Theft is always a possibility, especially in resource-constrained environments, as are the failure and misuse of a device.

Technological challenges are only a small contributor to the overall failures in data collection and digitization. Context of the venture is of primary concern as CHW programs vary from country to country.^17^ Local infrastructure and resources play a large role, as electricity and Internet access can be spotty in areas. Utilizing mobile devices for data collection in this manner requires consideration on both Internet capabilities and the availability of charging locations. The acceptability and security of digitally recording patient health data is a context-specific issue in which customers may react negatively based on personal beliefs and experience. Aggregation and analysis of the data prove to be difficult for inexperienced workers, who constitute the majority of the CHWs. Furthermore, it is important to note the lack of unique citizen identifiers in developing nations. Naming tends to be fluid and can change based on the societal position of the individual. Age too is complex, as many do not remember a specific birth date, making customer identification extremely difficult, if not impossible.

Coordination of data collection efforts becomes complicated in developing countries. Data collection over a large territory by a large team necessitates rigid procedures. If a change is to be made, convincing employees to follow through is difficult once they are already dispersed across a wider geographical area. Having a standard for the procedure is important, but often language is a barrier due to the varying dialects in developing nations. In this context, training in data collection can also be ineffective if conducted in a short amount of time, an issue that is exacerbated for projects being conducted internationally. A last enormous obstacle to the collection of health data is a lack of incentive for CWHs to collect health data. Since a majority of CHWs are unpaid volunteers, they are not encouraged to collect data, and it is often difficult for them to see the merit of the project.

## Typology of Data Monetization Programs

One of the primary challenges to the collection of useful health data is a lack of incentives for the aggregation of that information. Existing technologies and programs that attempt to digitize health data are often capital-intensive and, as detailed before, suffer from a variety of challenges. As a result, the International Telecommunications Union estimates that after a 3-year period, only 10% of such data collection efforts are successful.^18^ Yet, with data sales that will top over $10 billion by 2020 in the United States,^19^ pharmaceutical sales in Africa set to reach $30 billion by 2016,^20^ and an ever-increasing need for data in resource-constrained settings, an opportunity exists to combat the many issues facing data collection. One of the primary methods of combatting these difficulties is incentivizing data collection through the monetization of the aggregation process. In low–middle-income countries alone, millions of dollars are spent annually to gather health-related data, and so tapping those resources to scale the collection of data is an ideal solution.^21^ In fact, often the most successful telemedicine programs that do collect data are those that are funded by governments and corporate sponsors, who then use the system in place to obtain data as well as achieve publicity through advertisements during consultations.^†^ However, it is not well understood how best to incentivize data collection, which is why several modes of monetizing health data to both validate the need for data as well as ensure that the data collection process is fundamentally sustainable have been devised.

### Data sales that are funded by governments, nonprofits, and pharmaceutical companies

As data collection efforts have received increasing attention, one of the primary methods of funding data aggregation has arisen through external organizations. These groups are then often able to use that data or resell it for large profits to other interested entities. A simple business model would entail a CHW that is paid a weekly stipend to collect data on his or her target community by the entity. Payment would be on a holistic rather than individual record basis to avoid falsification of patient information. This data would then be available to the funder for use in many of the applications described previously. In places such as Brazil, the government requires and funds its CHWs to collect geographic, demographic, and health information on their assigned communities.^22^ This produces data that can be used by organizations to aid specific projects or people.^23^ Companies attempting to gain market share in a region, such as pharmaceutical companies spreading throughout Africa, also benefit greatly from this information.

Such efforts have been hypothesized before as a way of making data collection sustainable.^24^ This aggregated data can then be sold, as in many developed nations where the revenue stream funding the collection comes from data use in marketing and product sales.^25^ Health risk appraisal data can be utilized for focused marketing of health products. Data that is linked in any way to health behavior, such as medication usage patterns, is also a hot commodity for many pharmaceuticals.

International health operations such as Partners in Health (PIH) have had plans to commercialize their patient data previously, meaning that patient data outside of the normal scope of health information has some market. Similarly, Partners Healthcare, which operates the Mass General and Brigham & Women's hospitals in Boston, planned to sell their patient data to pharmaceutical and insurance companies as a source of revenue. This would also serve as a perfect opportunity for those companies to better understand the current state of healthcare for alteration of their business models and products.^26^ If a small portion of the data sales are returned to the original data collectors, particularly in developing nations, many of the challenges of incentivized data collection could be easily addressed. For the institutions funding the data aggregation, increased data points can result in more efficient operations, which reduces costs associated with wasted time and resources. While they might not necessarily see a direct profit, unless they sell the data, a decrease in wasted resources would actually more than fund their data collection efforts and result in money savings.

### Market data collection efforts to entities who pay for the collection of specific health information

An alteration to the model of organizational funding is that of a system where a health venture collects data specifically requested and funded by an entity. This model allows the organizations purchasing the data to have a greater role in the information that will be collected, which yields better-targeted resources and research. An established e-health venture could approach a nonprofit that works specifically with malnutrition about the possibility of tracking malnutrition severity and current intervention strategies. Such nonprofits likely deal with either large population sizes and are not well equipped to track all of their target populations, or are small and understaffed. Providing this company with a group of individuals who can, for a nominal fee, be paid to monitor their customers for them presents a win–win scenario for both parties.

One facet of the program in Peru that collects data on TB patient health is funded by the Peruvian government in collaboration with several other international organizations such as the WHO, CDC, and South African National TB program.^8^ This is an example of a service that collects specific health information as requested by a consortium of organizations all attempting to obtain a broader understanding of TB patient health. Similarly, in Rwanda, PIH pays its CHWs to collect household data on HIV and TB patients and their medication usage through a performance-based financing model.^27^ This helps them to better monitor their patients and ensures that funds are not wasted on unnecessary drugs and implementation efforts. Such funding entities are interested in a particular aspect of patient information, such as a count of patients with malaria in a region, which can allow that organization to determine the needs of the specific patients as well as the overall needs of a community.^28^ Additionally, workers like CHWs stand to gain an extra revenue source while fulfilling some of their primary goals of advocating for the health of local communities.^29^

Government and nonprofit data collection efforts are also widespread, existing throughout the developing world. Demographic and Health Surveys (DHS) is a USAID-funded data collection and dissemination effort that monitors and evaluates population-level health and nutrition programs in over 80 countries across the globe, including 46 alone in Africa.^30^ The program works in tandem with partners such as Johns Hopkins University, and conducts surveys at the request of a USAID mission or international donor in a country that receives U.S. foreign aid. The organization works with local stakeholders to collect data and also leaves behind an increased capacity for the aggregation of high-quality data in the host country. Another specific data collection venture is Mapping Malaria Risk in Africa, which maps malaria risk in Africa through funding from aid grants and sells the data on a per country basis.^31^

Efforts by pharmaceutical companies examining health and demographic information exist in developing countries as well. These companies often utilize local workers to collect data that can be used to help market and brand drugs.^20^ CHWs can help to support assessments of payment channels and patient preferences relevant to the pharmaceutical companies' operations. Patient health data is in fact so important to pharmaceutical companies, that in 2004, the AMA Board of Trustees noted that the companies “would struggle without the data, resulting in…less targeted educational information, and fewer or less relevant drug samples. The public good uses of the data would be severely cut back or eliminated as [healthcare information organizations] would no longer have a financial incentive to maintain the data.”^32^

### Creation of health database that can be accessed for a fee by interested parties

While more traditional methods of monetizing and implementing data aggregation processes have existed for decades, a more recent novelty is the compilation of health information databases that can either be purchased or accessed for a subscription fee by consumers. Independent Health Record Banks are databases of this nature that give medical information consumers a place to purchase patient data for uses related to their own operations.^33^ The databases are patient owned, similar to credit unions, so that any data sales that occur are passed back as a financial benefit to the data owners. It has even been theorized, although not implemented, that any revenue could be credited to the data owner's “account” and then withdrawn, similar to a bank. With a venture such as Mashavu: Networked Health Solutions in Nyeri, Kenya, which gathers over 30,000 customer data points annually, a database could be compiled on the specific town in which operations are conducted. This database would then be available to the Kenyan government or nonprofits in the region for a small fee that would be utilized to fund further CHW-driven data collection efforts.

Database sales have been proven to be financially stable, as illustrated by Euromonitor International, which sells published reports on consumers and demographics in over 50 countries at prices upward of $900 per report.^34^ Organizations that purchase aggregated data from individuals or countries are able to sell it as de-identified patient data for billions of dollars annually, such as IMS Health, which sells over $2 billion of data from at least 100 different countries.^35^ The American Medical Association, which talks of the importance of data to pharmaceuticals, received over $44.5 million (16% of their total revenue) in 2005 from database sales.^36^ If the financial compensation received for data sales is then turned around to fund further data collection efforts, it creates a sustainable loop that allows all parties to benefit.

### Subscription fee for accessing patient medical history

A less explored model of incentivizing data collection entails the storage of patient health history to track health trends over time, but then charging customers a fee for accessing their long-term health information. Such a model would prove to be sustainable and would give patients further insight into their medical records. A subscription could be monthly or annual, and could be used to drastically improve quality of life for much of the population as individuals can anticipate and prevent future health problems. CHWs or other healthcare workers who collect data could use a mobile connection to access digitally stored data for a patient and would then be paid a small fee for the service by the customer. This fee, over a larger number of individuals, would be able to fund all data collection efforts to keep that aspect of the health venture sustainable. The patient health information could also be easily printed for the patients for a separate fee, which is particularly important if a patient needs to visit a health clinic. The nurses and doctors could use this past medical information to better address health problems and prescribe medications. The fee for a printout of a patient's medical history would further contribute to the sustainability of data collection and allow the wide-scale aggregation of data for a greater understanding of public health.

## Conclusions

While there is no question that big data and machine learning would have a large impact on the improvement of healthcare in developing countries, there is still much work to be done. Creating an infrastructure to support data collection is the first step. There are many challenges to the collection of reliable data, one of the biggest ones being a lack of clear incentive mechanisms and business models that enable CHWs to devote time and energy toward this purpose. However, the success of such projects hinges on more than just economic incentives. Creating a culture, both among the CHWs and the general public, that values data collection and has knowledge of the impact it can have would go a long way to comprehensive improvements in health services and systems.

CHWs, as trusted and respected members of their communities, are fundamentally interested in the improvement of their community's health. Providing them with evidence, in the form of decreased clinic wait-times or less-expensive drugs for example, coupled with an incentive structure, is essential. This can lead to further large-scale buy-in from their fellow community members that will in-turn increase reliability and accuracy of collected information. Data collection programs create value at many levels of society, from governments concerned about the care of their citizens, to CHWs who may be struggling economically, to community members demanding better healthcare. Creating and strengthening the data collection infrastructure and incentivizing those bridging the last mile data collection gaps opens the door for big data scientists and machine learning experts to ultimately realize the potential of leveraging data for social good.

## Notes

^†^ Sundin P, Callan J, Mehta K. Why do entrepreneurial mHealth ventures in the developing world fail to scale? ASME Demand (In Review).

## Abbreviations Used

CHWs: community health workers
D4D: Data for Development
GIVS: Global Immunization and Vision Strategy
IDS: integrated disease surveillance
PIH: Partners in Health
